# Microbiota Regulates Phosphatidylcholine in the Intestinal Mucus Lipidome of Mice in a Strain‐ and Segment‐Dependent Manner

**DOI:** 10.1002/edm2.70380

**Published:** 2026-11-10

**Authors:** Mia Øgaard Mønsted, Mesut Bilgin, Lukasz Krych, Emma Schack Wiedenbein, Camilla Hartmann Friis Hansen, Martin Haupt‐Jorgensen

**Affiliations:** ^1^ Department of Pathology, The Bartholin Institute Rigshospitalet Copenhagen Denmark; ^2^ Lipidomics Core Facility Danish Cancer Institute Copenhagen Denmark; ^3^ Department of Food Science, Faculty of Science University of Copenhagen Frederiksberg Denmark; ^4^ Department of Veterinary and Animal Sciences, Faculty of Health and Medical Sciences University of Copenhagen Frederiksberg Denmark

**Keywords:** antibiotics, germ‐free mice, intestinal barrier dysfunction, mucus lipids, phosphatidylcholine, type 1 diabetes

## Abstract

**Background:**

Type 1 diabetes (T1D) is associated with intestinal barrier dysfunction, which depends on an intact mucus layer. Phosphatidylcholine (PC) is a major lipid of intestinal mucus that likely exerts an important barrier function due to its hydrophobic and anti‐inflammatory properties. Recently, we showed that PC is reduced in jejunal and colonic mucus in pre‐diabetic non‐obese diabetic (NOD) mice compared to C57BL/6 (B6) mice. As such, PC may play a protective role in T1D. Some bacteria produce PC. However, it is unknown whether T1D‐associated dysbiosis (i.e., altered microbial composition) is behind the reduced PC content in the intestine.

**Methods:**

Here, we examined the impact of gut microbiota on jejunal and colonic mucus PC composition. This was done by treating 4 weeks old pre‐diabetic NOD and B6 mice with a broad‐spectrum antibiotic cocktail and by breeding germ‐free BALB/c mice, which were then compared with untreated barrier‐housed NOD, B6 and BALB/c mice. The intestinal mucus lipid profile was analysed by quantitative shotgun lipidomics, while the relative composition of the faecal microbiota was assessed by 16S rRNA gene amplicon sequencing and verified by absolute quantifications using qPCR.

**Results:**

Comparing jejunum and colon from the mice revealed that the overall intestinal mucus lipidome was segment‐ but not strain‐specific. Antibiotic treatment reduced colonic PC levels in the NOD and B6 mice, whereas germ‐free conditions did not in the BALB/c mice, which, independent of the presence of microbiota, had the lowest levels of colon PC. The faecal microbiome composition also differed between the three strains of untreated barrier‐housed mice, including reduced abundance of phyla with PC‐containing bacteria in the BALB/c mice.

**Conclusions:**

Results suggest that gut microbiota perturbation is an important determinant of intestinal mucus PC content, although the effect seems to depend on the host‐specific baseline microbiota composition.

## Graphical abstract

Gut microbiota perturbation alters the intestinal mucus lipidome, with reduced phosphatidylcholine (PC) levels in colonic mucus and changes in lipid species in jejunal mucus.

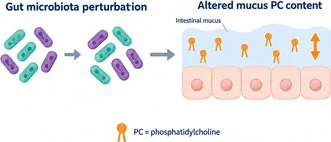

## 1 Introduction

The intestinal barrier function in both pre‐ and manifest type 1 diabetes (T1D) is aberrant and involves increased intestinal permeability and a skewed microbial composition [1, 2]. Moreover, T1D progressors with ≥ 2 islet autoantibodies have significantly increased blood lactulose:rhamnose ratio (small intestinal permeability) compared to non‐progressors with ≥ 2 islet autoantibodies [3], indicating that intestinal permeability could be involved in T1D progression. Potential disease mechanisms caused by increased intestinal permeability include increased flux of diabetogenic microbial and food molecules from the intestinal lumen to the body, which may activate autoreactive T cells via molecular mimicry [4] or beta‐cell stress [5] causing beta‐cell autoimmunity and T1D in genetically predisposed individuals. Thus, the intestinal barrier function is a promising therapeutic target.

The intestinal mucus layer is the first line of defence against luminal molecules. It consists of mucins, water, lipids, electrolytes and other proteins important for its structure and function [6]. Little is known about the role of the intestinal mucus lining in T1D; however, recent studies found reduced levels of goblet cells and *Muc2* expression in both small and large intestinal tissue of pre‐diabetic and diabetic non‐obese diabetic (NOD) mice compared to control strains [7, 8, 9]. Recently, we discovered that the level of phosphatidylcholine (PC), a major mucus lipid, is reduced in jejunal mucus of pre‐diabetic NOD mice versus age and diet matched C57BL/6 (B6) mice [10]. The study also showed reduction of specific PC species in colonic mucus at different pre‐diabetic ages in the NOD mice. This may contribute to intestinal barrier dysfunction in T1D due to the hydrophobic and anti‐inflammatory properties related to PC [11, 12, 13, 14, 15]. In support of this notion, NOD mice have a reduced intestinal permeability compared to B6 mice at ages 16–18 weeks [7]. Moreover, our recent study showed that the β‐diversity of the cecal microbial community differed between the NOD and B6 mice [10], which might have affected the mucus lipid composition, since other studies showed that the microbiota composition can modulate goblet cell numbers and penetrative properties of the mucus layer [8, 16]. Also, it is estimated that 10% of all bacteria produce PC [17]. Therefore, we hypothesized that perturbation of the gut microbiota modifies host‐derived PC composition in the intestinal mucus layer.

Here we examined the impact of intestinal microbiota on mucus lipid composition in the jejunum and colon by comparing three mouse strains that were either fully colonized, barrier‐housed, treated with a cocktail of broad‐spectrum antibiotics or bred germ‐free.

## 2 Materials and Methods

### 2.1 Animals

The animal experiment was approved by the Danish Animal Experiments Inspectorate (licence number 2017‐15‐0201‐01263) and adhered to the principles of the Directive 2010/63/EU of the European Parliament and the Council of 22nd September 2010 on the protection of animals used for scientific purposes and the Danish Animal Experimentation Act (LBK 474 15/05/2014).

NOD/MrkTac (NOD), C57BL/6J (B6) and BALB/cAnNTac (BALB/c) (All from Taconic, Lille Skensved, Denmark) mice were bred and housed under standard conditions (12 h light cycle, temperature 22°C, humidity 55% ± 10%) in open cages in a barrier‐protected animal facility (Faculty of Health and Medical Sciences, University of Copenhagen, Frederiksberg, Denmark). Hence, all conventionally housed mice were obtained from the same vendor and maintained in the same animal facility. Mice had free access to bottled tap water and Altromin 1324 chow (Brogaarden, Lynge, Denmark). Pups from a minimum of two different litters were used in each experimental group. Only female offspring were used in the study.

### 2.2 Microbiota Depletion

To assess the effect of the gut microbiota on intestinal mucus lipid composition, the microbiota was depleted by treating NOD mice and B6 mice with an antibiotic cocktail (Figure 1a). The cocktail consisted of 1 g/L ampicillin, 0.5 g/L vancomycin and 1 g/L neomycin in tap water, which was changed twice a week. The treatment started when mice were visibly pregnant (around 2 weeks of gestation) and continued until the experiment ended (offspring aged 4 weeks). In addition, germ‐free BALB/c mice were bred and housed in an AAALAC‐accredited germ‐free facility in high‐efficiency particulate air‐ventilated flexible film isolators (PFI Systems, Milton Keynes, United Kingdom) (12 h light cycle; pressure, 110 Pa; 23°C). The germ‐free mice had free access to sterile water and irradiated Altromin 1324 chow (Brogaarden). Mice were not weaned before being killed at 4 weeks of age. Female offspring from two different litters were used.

**FIGURE 1:**
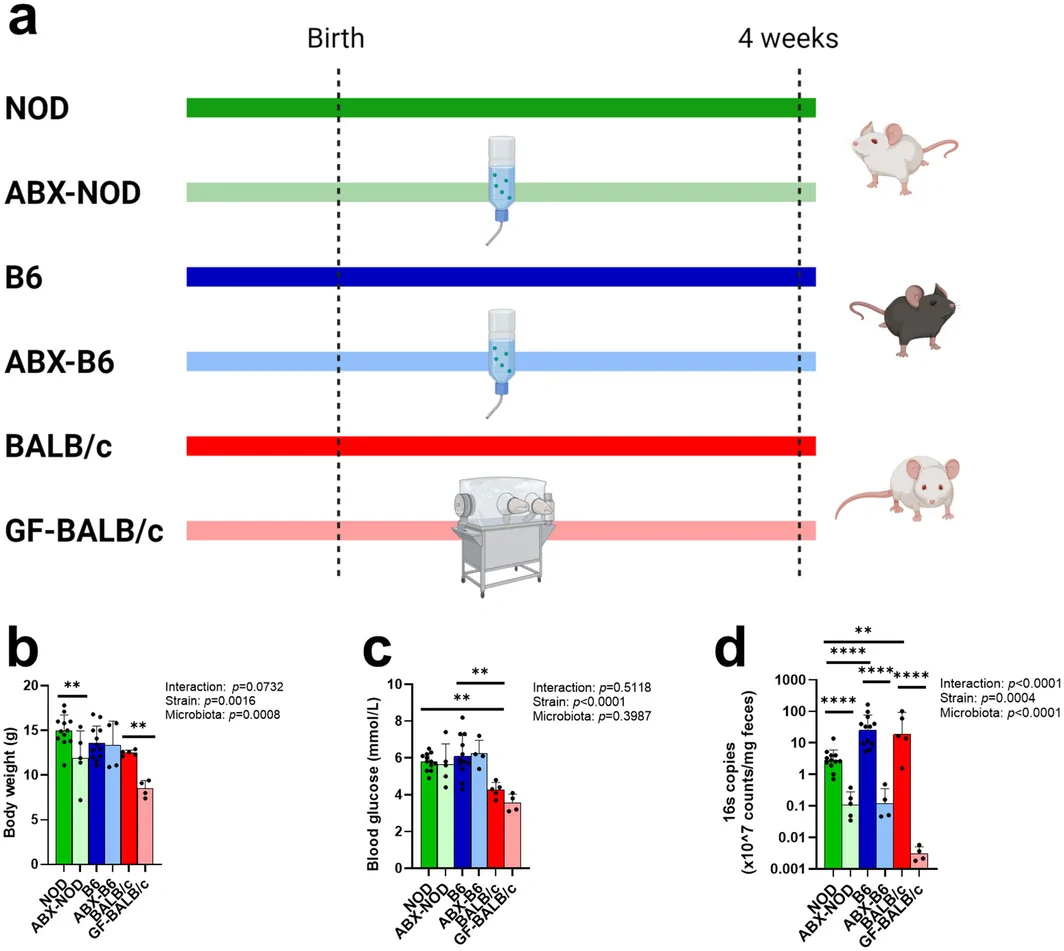
Effect of antibiotic treatment. (a) Experimental set‐up. Non‐fasted body weight (b) and blood glucose (c) at 4 weeks of age. Total number of bacterial 16s copies per mg of faeces from 4‐week‐old mice (d). *n* = (b–d) NOD: 12, ABX‐NOD: 5, B6: 12, ABX‐B6: 4, BALB/c: 5; (b–d) GF‐BALB/c: 4. Dark colours = microbiota presence, light colours = microbiota absence; green = NOD (NOD/ABX‐NOD), blue = B6 (B6/ABX‐B6), red = BALB/c (BALB/c/GF‐BALB/c). Statistical analysis was performed with 2‐way ANOVA fitting a full model (interaction plus effect of the two main effects [mouse strain and gut microbiota presence]) and individual multiple comparisons between all groups using Šidák correction (c, d). Log transformation was performed on (d) prior to analysis. Data are represented as means ± SD. **p < 0.01; ****p < 0.0001.

### 2.3 Experimental Animal Procedures

At 4 weeks of age, mice were weighed and blood glucose was measured on venous tail blood using the Freedom Lite blood glucose meter (Abbott Diabetes Care, Alameda, CA). Then, mice were placed in clean empty cages and faecal pellets collected and immediately snap frozen in liquid nitrogen. Mice were then fasted for 4 h with free access to bottled tap water and killed by cervical dislocation. The duodenojejunal flexure was used to distinguish duodenum and proximal jejunum. Faeces were gently removed from proximal jejunum and colon until macroscopically clean. Longitudinal incisions were made in the intestinal segments, and mucus gently collected with a metal spatula to minimize contamination with epithelial cells, followed by snap freezing.

### 2.4 DNA Purification

Faecal samples were thawed on ice, and DNA was extracted using the Bead‐Beat Micro AXE Gravity kit (A&A Biotechnology, Gdańsk, Poland) according to the manufacturer's protocol. DNA concentration and purity were determined using a NanoDrop ND‐1000 spectrophotometer (Saveen and Werner AB, Limhamn, Sweden).

### 2.5 qPCR

To assess the efficacy of antibiotic treatment and confirm germ‐free status, qPCR targeting the V3 region of the 16S rRNA gene was performed on faecal DNA. The qPCR was run on a 7500 Fast real‐time PCR instrument (Applied Biosystems, Foster City, CA) using 100 ng DNA template, primer PRBA388F (5′‐ACWCCTACGGGWGGCAGCAG‐3′) and PRUN518R (5′‐ATTACCGCGGCTGCTGG ‐3′), and SYBR green PCR Master Mix (Applied Biosystems) with qPCR conditions as described previously [18]. *Escherichia coli K*‐12 was used as a positive control and standard [19]. Quantified 16S rRNA values were normalized to the weight of the original sample. The DNA concentration in negative control samples was subtracted from faecal DNA concentrations.

### 2.6 Lipidomics

Lipid extraction of mucus sample homogenates (35 μg total protein in 200 μL 155 mmol/L ammonium bicarbonate) was performed on ice by a modified Bligh and Dyer protocol [20, 21, 22]. Samples were spiked with 12.5 μL internal lipid standard mix (composition described in [10]). Subsequently, 1 mL chloroform/methanol (2:1, v/v) was added, tubes shaken (2000 rpm, 20 min, 4°C) and centrifuged (500 g, 1 min, 4°C). Phase separation resulted in an upper aqueous phase and a lower organic phase (lipid‐containing). The organic phase was vacuum evaporated following mass spectrometry (MS) analysis using a Q‐Exactive (Quadrupole‐Orbitrap Mass Spectrometer) coupled to a robotic sample infuser (TriVersa NanoMate, Advion Biosciences, Ithaca, NY). LipidXplorer and LipidQ were used for data processing [20, 23, 24]. Annotation of the lipid species was previously described [20, 25], and cardiolipin (CL) and triacylglycerols (TAG) were not considered.

Sterol lipid (ST), glycerolipid (GL) and glycerophospholipid (GPL) species were annotated by ‘species level’ as follows: <lipid class> <total number of carbon atoms in fatty acyl moieties>:<total number of double bonds in fatty acyl moieties> (e.g., PC 34:1). Ether‐linked species are denoted by the prefix ‘O‐’ (e.g., PC O‐34:1). Sphingolipid (SL) species were annotated by ‘species level’ as follows: <lipid class> < total number of carbon atoms in the long‐chain base and fatty acyl moiety>:<total number of double bonds in the long‐chain base and fatty acyl moiety>;<total number of OH groups in the long‐chain base and fatty acyl moiety> (e.g., SM 34:1;2). Two mucus samples were removed due to mismarked labels. Table S1 shows the lipid class distribution in jejunal and colonic mucus, and Table S2 shows the lipid species distribution.

### 2.7 Microbiota

Relative abundance of the bacterial community was evaluated using nanopore‐based sequencing of the near full 16S rRNA gene amplicons using GridIONx5 (Oxford Nanopore Technologies, Oxford, UK), as previously described [26]. The raw FASTQ data were analysed using the Laca pipeline [27]. The generated abundance table was further analysed using the Qiime2 (v2018.11) bioinformatic platform [28]. Alpha (observed species index) and beta (PCoA based on Bray‐Curtis and Jaccard distance matrices) diversity were calculated using rarefied feature tables (8000 reads per sample). Permutational multivariate analysis of variance (PERMANOVA) was used to test differences between the three groups based on Bray‐Curtis and Jaccard distance matrices. The differences in taxa relative abundance were analysed with Analysis of composition of microbiomes (ANCOM) [29].

### 2.8 Statistics

Statistical analyses were performed in GraphPad Prism version 9.3.1 (La Jolla, CA). Two‐way ANOVA with Šidák post hoc test was applied, grouping the data based on microbiota presence and strain. When the assumptions for two‐way ANOVA were not met, log or square transformations were performed to achieve residual normality and to eliminate outliers (identified using the ROUT method with *Q* = 1%). Principal component analysis (PCA) was conducted to detect the largest variance in the lipidomic data sets from jejunal and colonic mucus, with principal components selected based on parallel analysis at the 95th percentile level with 1000 simulations. Lipid species with zero abundance were set to 0.0001% (detection limit). Multiple unpaired *t*‐tests were performed using the two‐stage step‐up method of Benjamini, Krieger and Yekutieli, with a false discovery rate of 5%. For all data, *p*‐ or *q*‐values < 0.05 were considered statistically significant, while *p*‐values < 0.10 were considered indicative of a trend (not shown in graphs for two‐way ANOVA analyses).

## 3 Results

### 3.1 Effective Depletion of Intestinal Microbiota

Antibiotic treatment and the germ‐free conditions significantly reduced the body weights of the 4‐week‐old NOD mice and BALB/c mice; however, not for the B6 mice (Figure 1b). The interventions did not change the non‐fasting blood glucose, but it was reduced in BALB/c mice compared to NOD and B6 mice regardless of the presence of a microbiome (Figure 1c). The depletion of intestinal microbiota was effective in NOD, B6 and BALB/c mice as seen from the reduced number of faecal 16S rRNA gene copies, though the antibiotics failed to deplete the microbiome to a level comparable to the germ‐free mice (Figure 1d). Moreover, untreated NOD mice had reduced faecal 16S rRNA gene copy numbers compared to untreated B6 and BALB/c mice (Figure 1d).

### 3.2 Spatially Distinct Lipid Profile in Jejunal and Colonic Mucus

A PCA analysis of the intestinal mucus lipid profile revealed that jejunal and colonic samples clustered distinctly regardless of strain and microbiota presence (Figure 2a). This spatial (jejunum vs. colon) difference in lipid composition was also observed on species level, as distinct jejunal and colonic lipid patterns were identified (Figure 2b). Next, we tested whether the presence of microbiota changed the number of detected lipid species in jejunal and colonic mucus. In jejunal mucus, an average of 226 ± 16 lipid species were detected in the mice altogether and with the highest levels observed in BALB/c mice (Figure 2c). Interestingly, the antibiotic treated B6 mice and the germ‐free BALB/c mice had significantly reduced numbers of lipid species compared to their untreated controls, whereas the antibiotic treatment had no effect on the NOD mice (Figure 2c). In colonic mucus, an average of 288 ± 14 lipid species were detected in the mice altogether; thus, a higher number of lipid species than found in jejunal mucus, showing that jejunal and colonic mucus have distinct lipid species profiles and differ in lipid quantities. No significant differences were observed between any of the mouse groups in the colonic lipid species number (Figure 2h). Detected lipid classes in jejunal and colonic mucus are depicted in Figure 2d–g and Figure 2i–l, respectively, where especially specific spatial differences were observed in the alkyl‐PC (PCO‐), phosphatidylethanolamine (PE), alkyl‐PE (PEO‐), lysophosphatidylcholine (LPC) and sphingomyelin (SM) levels between the two intestinal segments.

**FIGURE 2:**
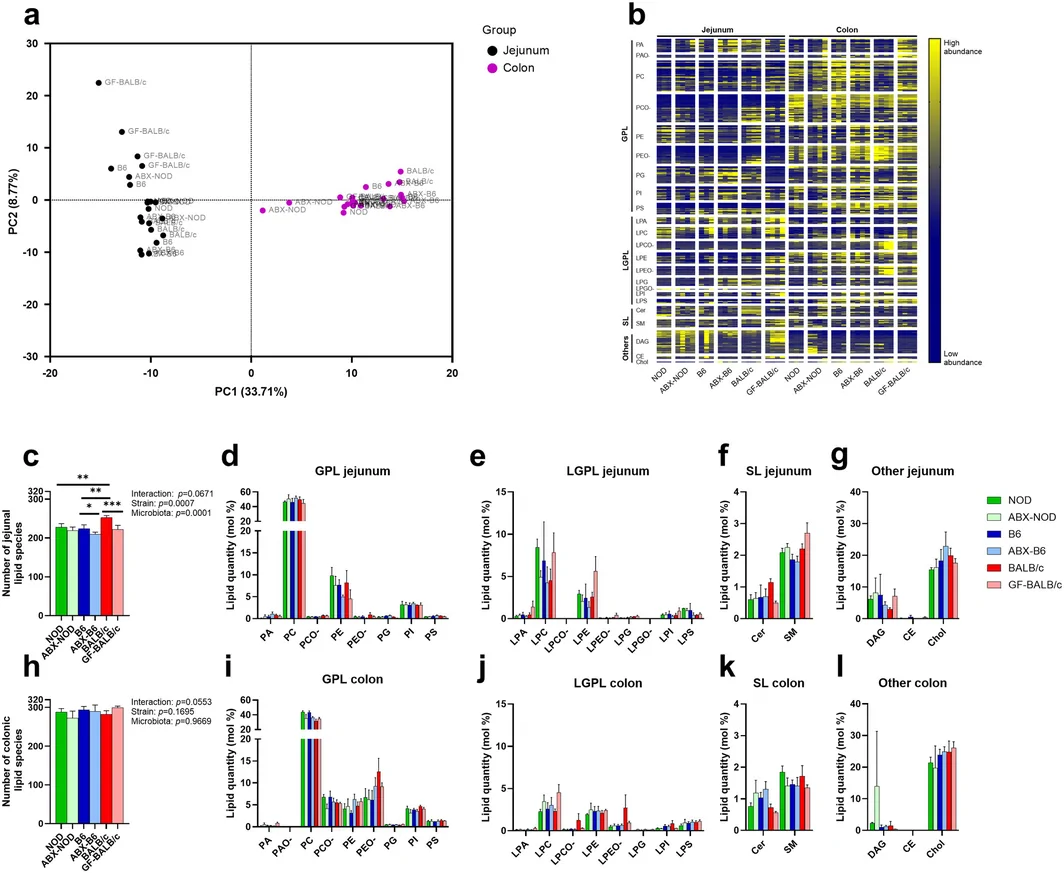
Lipid composition of jejunal and colonic mucus. (a) PCA plot visualizing the largest variations (PC1 and PC2) in the data comprised of all detected lipid species in jejunum and colon in all mouse groups. (b) Heatmap visualizing the abundance of lipid species between all samples. Rows were standardized by subtracting row mean and dividing by SD. The data were grouped based on lipid classes (*y*‐axis), mouse groups (*x*‐axis) and intestinal segments (*x*‐axis) where each lipid species were sorted from high to low based on absolute loading scores from PC1 of the PCA. Colour scale was based on 5% and 95% percentiles to avoid overrepresenting outliers. Number of detected lipid species in jejunal (c) and colonic (h) mucus. Lipid quantity of all lipid classes in jejunal (d–g) and colonic (i–l) mucus. The lipid classes were grouped into glycerophospholipids (GPL) (d, i) lysoglycerophospholipids (LGPL) (e, j), sphingolipids (SL) (f, k) and the remaining classes as ‘others’ (g, l). *n* = (c–l) NOD: 3, ABX‐NOD: 4, B6: 3, ABX‐B6: 4, BALB/c: 4, GF‐BALB/c: 4. (a) black = jejunal mucus samples, purple = colonic mucus samples, (b) yellow = high abundance, dark blue = low abundance, (c–l) Dark colours = microbiota presence, light colours = microbiota absence; green = NOD (NOD/ABX‐NOD), blue = B6 (B6/ABX‐B6), red = BALB/c (BALB/c/GF‐BALB/c). Statistical analysis was performed with 2‐way ANOVA fitting a full model (interaction plus effect of the two main effects [mouse strain and microbiota presence]) and performing individual multiple comparisons between all groups using Šidák correction (c, h). *y*‐squared transformation was performed on (h) prior to analysis. (c–l) Data are represented as means ± SD. *p < 0.05; **p < 0.01; ***p < 0.001.

### 3.3 Reduced Colonic Phosphatidylcholine Class Quantity After Antibiotic Treatment

The impact of the microbiota on mucus PC quantity was evaluated for jejunal and colonic mucus. In jejunal mucus, the quantity of the PC class as a whole was not altered between the groups (Figure 3a). As also found in [10], the most abundant PC species in jejunal mucus were PC 34:2 (17.9 ± 2.7 mol%), PC 36:2 (14.1 ± 3.0 mol%), PC 36:3 (2.3 ± 0.5 mol%), PC 36:4 (5.4 ± 0.7 mol%) and PC 38:4 (3.2 ± 0.6 mol%) (Figure 3b). In colonic mucus, the PC levels were significantly reduced in antibiotic‐treated NOD mice and B6 mice compared to their untreated controls whereas the germ‐free housing had no impact in the BALB/c mice (Figure 3c). Importantly, the level of PC in colonic mucus of untreated barrier‐housed BALB/c mice was already at baseline at a significantly reduced level compared to the untreated NOD and B6 mice, to a level comparable to the germ‐free and antibiotic‐treated mice (Figure 3c). The most abundant PC species in colonic mucus were PC 34:1 (6.0 ± 1.6 mol%), PC 34:2 (6.9 ± 1.2 mol%), PC 36:2 (4.5 ± 1.1 mol%), PC 36:3 (4.1 ± 0.6 mol%), PC 36:4 (3.4 ± 0.7 mol%) and PC 38:4 (1.9 ± 0.4 mol%) (Figure 3d), which is in accordance with our recent study [10].

**FIGURE 3:**
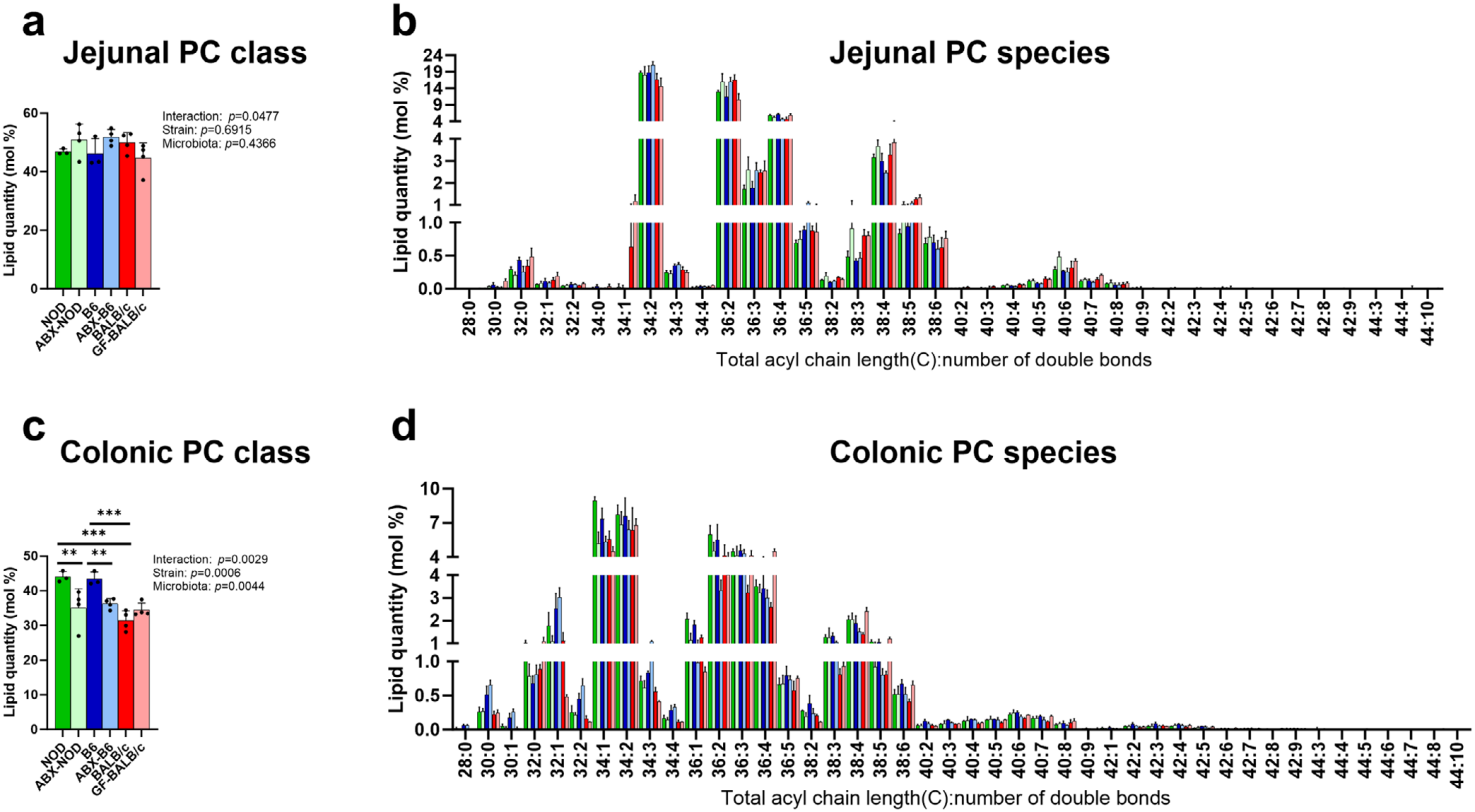
PC content in jejunal and colonic mucus. Lipid quantity of the PC class in jejunal mucus (a) and colonic mucus (c). Lipid quantity of all detected PC species in jejunal mucus (b) and colonic mucus (d). *n* = (a–d) NOD: 3, ABX‐NOD: 4, B6: 3, ABX‐B6: 4, BALB/c: 4, GF‐BALB/c: 4. Dark colours = microbiota presence, light colours = microbiota absence; green = NOD (NOD/ABX‐NOD), blue = B6 (B6/ABX‐B6), red = BALB/c (BALB/c/GF‐BALB/c). Statistical analysis was performed with 2‐way ANOVA fitting a full model (interaction plus effect of the two main effects [mouse strain and microbiota presence]) and performing individual multiple comparisons between all groups using Šidák correction. (a–d) Data are represented as means ± SD. **p < 0.01; ***p < 0.001.

### 3.4 Altered PC Species Distribution in Jejunal Mucus of BALB/c Mice

The number of jejunal mucus lipids was lower in the microbiota depleted mice (Figure 2c), suggesting that bacterial lipids may contribute to the lipid species composition in jejunal mucus. However, antibiotic treatment did not affect the abundance of PC species specifically (Figure 4d,e). Only in the BALB/c mice, the abundance of specific PC species showed microbiota dependence (Figure 4f), possibly because of more effective microbial eradication compared to antibiotic treatment in the other strains (Figure 1d). There were also clear differences in the abundance of specific PCs between the mouse strains, independently of the presence of microbes (Figure 4b,c,h). In particular, the BALB/c mice diverged from the other strains.

**FIGURE 4:**
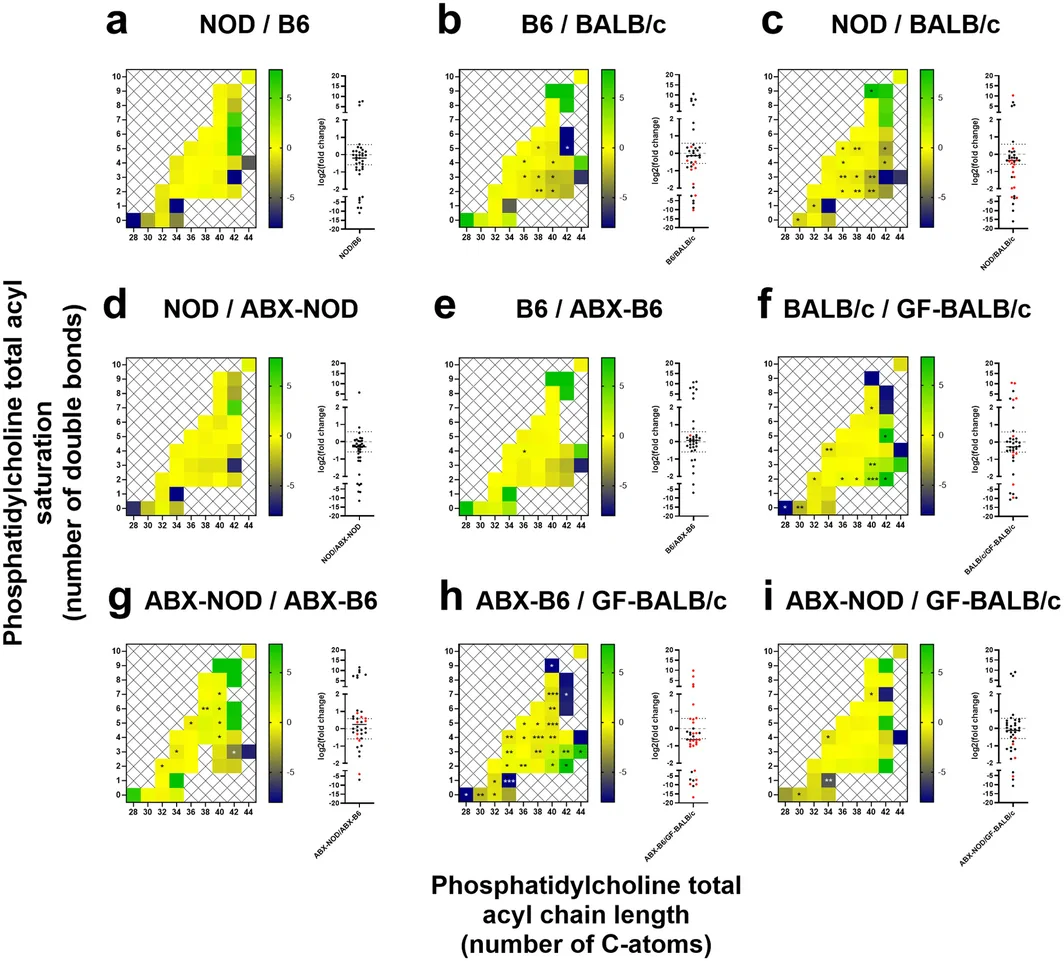
Strain and microbiota effects on jejunal PC species. Heatmap visualizing the log_2_ (fold change) between PC species in jejunal mucus when comparing NOD versus B6 (a) B6 versus. BALB/c (b) NOD versus BALB/c (c), NOD versus ABX‐NOD (d), B6 versus ABX‐B6 (e) BALB/c versus GF‐BALB/c (f) ABX‐NOD versus ABX‐B6 (g) ABX‐B6 versus GF‐BALB/c (h) and ABX‐NOD versus GF‐BALB/c (i) Each square represents the log_2_ (fold change) of the specific PC species with fatty acids of *x* length and *y* number of double bonds. Colour scale was based on 5% and 95% percentiles of log_2_ (fold change)‐values to avoid overrepresenting outliers. Green—yellow—dark blue = increased quantity—no difference –decreased quantity in the first‐mentioned compared to the last‐mentioned group (e.g., NOD in NOD/B6 or ABX‐B6in ABX‐B6/GF‐BALB/c). For easier visualization, a linear log_2_ (fold change) graph is included beside each heatmap representing the same changes between groups; however, highlighting general increases/decreases in log_2_ (fold change) between the groups. *n* = (a, c, d) NOD: 3, (d, g, i) ABX‐NOD: 4, (a, b, e) B6: 3, (e, g, h) ABX‐B6: 4, (b, c, f) BALB/c: 4, (f, h, i) GF‐BALB/c: 4. Statistical analysis was performed with multiple unpaired *t*‐testing with 5% FDR (two‐stage step‐up [Benjamini, Krieger and Yekutieli] method). For heatmaps: *p < 0.05; **p < 0.01; ***p < 0.001, for linear fold change plots: Significant different (*p* < 0.05) changes are marked with red colour.

### 3.5 Altered PC Species Distribution in Colonic Mucus of BALB/c Mice

Differences in the abundance of specific PC species in colonic mucus, as for jejunum, were primarily observed comparing the germ‐free BALB/c group with its microbiota competent control group (Figure 5f) and the BALB/c groups with the other strains (Figure 5b,c,h). Our recent study found that the levels of many PC species were reduced in colonic mucus of NOD mice compared to B6 mice [10]. In the current study, we observed many similar tendencies, although mostly non‐significant (Figure 5a). Again, the antibiotic treatment did not significantly alter the PC species composition in colon (Figure 5d,e), possibly due to less effective microbiota eradication compared to the germ‐free BALB/c mice (Figure 1d).

**FIGURE 5:**
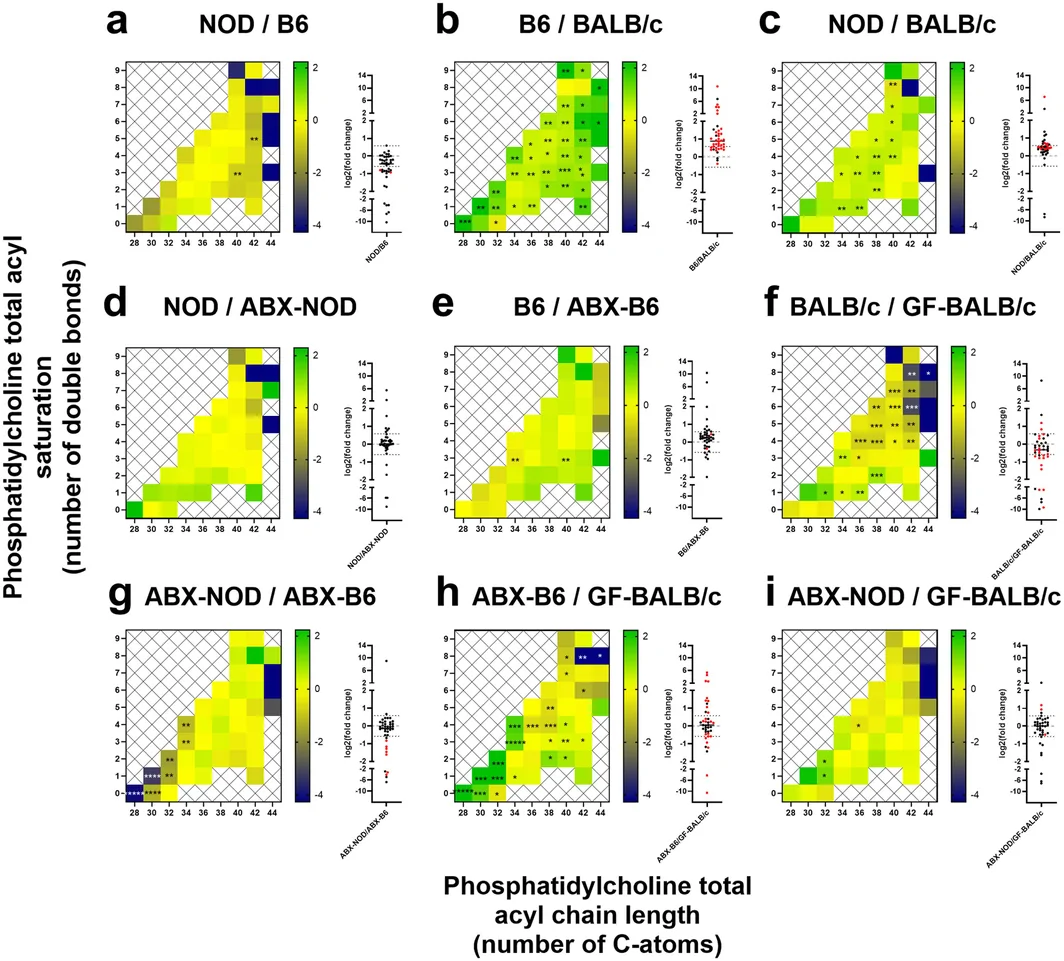
Strain and microbiota effects on colonic PC species. Heatmap visualizing the log_2_ (fold change) between PC species in colonic mucus when comparing NOD versus B6 (a) B6 versus BALB/c (b) NOD versus BALB/c (c) NOD versus ABX‐NOD (d), B6 versus ABX‐B6 (e), BALB/c versus GF‐BALB/c (f) ABX‐NOD versus ABX‐B6 (g) ABX‐B6 versus GF‐BALB/c (h) and ABX‐NOD versus. GF‐BALB/c (i). Each square represents the log_2_ (fold change) of the specific PC species with fatty acids of *x* length and *y* number of double bonds. Colour scale was based on 5% and 95% percentiles of log_2_ (fold change)‐values to avoid overrepresenting outliers. Green—yellow—dark blue = increased quantity—no difference –decreased quantity in the first‐mentioned compared to the last‐mentioned group (e.g., NOD in NOD/B6 or ABX‐B6 in ABX‐B6/GF‐BALB/c). For easier visualization a linear log_2_ (fold change) graph is included beside each heatmap representing the same changes between groups; however, highlighting general increases/decreases in log_2_ (fold change) between the groups. *n* = (a, c, d) NOD: 3, (d, g, i) ABX‐NOD: 4, (a, b, e) B6: 3, (e, g, h) ABX‐B6: 4, (b, c, f) BALB/c: 4, (f, h, i) GF‐BALB/c: 4. Statistical analysis was performed with multiple unpaired *t*‐testing with 5% FDR (two‐stage step‐up [Benjamini, Krieger and Yekutieli] method). For heatmaps: *p < 0.05; **p < 0.01; ***p < 0.001, for linear fold change plots: Significant different (*p* < 0.05) changes are marked with red colour.

### 3.6 Differing Faecal Microbiota Composition Between Mouse Strains

To assess the association between mucus lipid composition and faecal microbiota composition, faecal microbial communities of untreated mice were analysed with 16S rRNA gene amplicon sequencing. Microbiota composition differed significantly between mouse strains (NOD, BALB/c and B6), both quantitatively (Bray–Curtis distance; PERMANOVA; pseudo‐*F* = 6.213, *p* = 0.001) (Figure 6a) and qualitatively (Jaccard distance; PERMANOVA; pseudo‐*F* = 2.987, *p* = 0.001) (Figure 6b). Across all strains, the dominant phylum by relative abundance was Firmicutes (48.5%–80.6%), followed by Bacteroidetes (17.7%–34.2%); the remaining phyla included Actinobacteria, *Candidatus* Saccharibacteria, Proteobacteria and Verrucomicrobia (Figure 6c; Table S3 for details). Relative abundances at the family level are shown in Figure 6d. ANCOM analysis revealed distinct microbial signatures across the three mouse strains: Muribaculaceae and Parasutterella were enriched in B6 mice, whereas Streptococcus, Coriobacteriaceae and Clostridium were more abundant in NOD mice. In contrast, Eubacterium was elevated in BALB/c mice, highlighting strain‐specific differences in gut microbiota composition (Figure 7).

**FIGURE 6:**
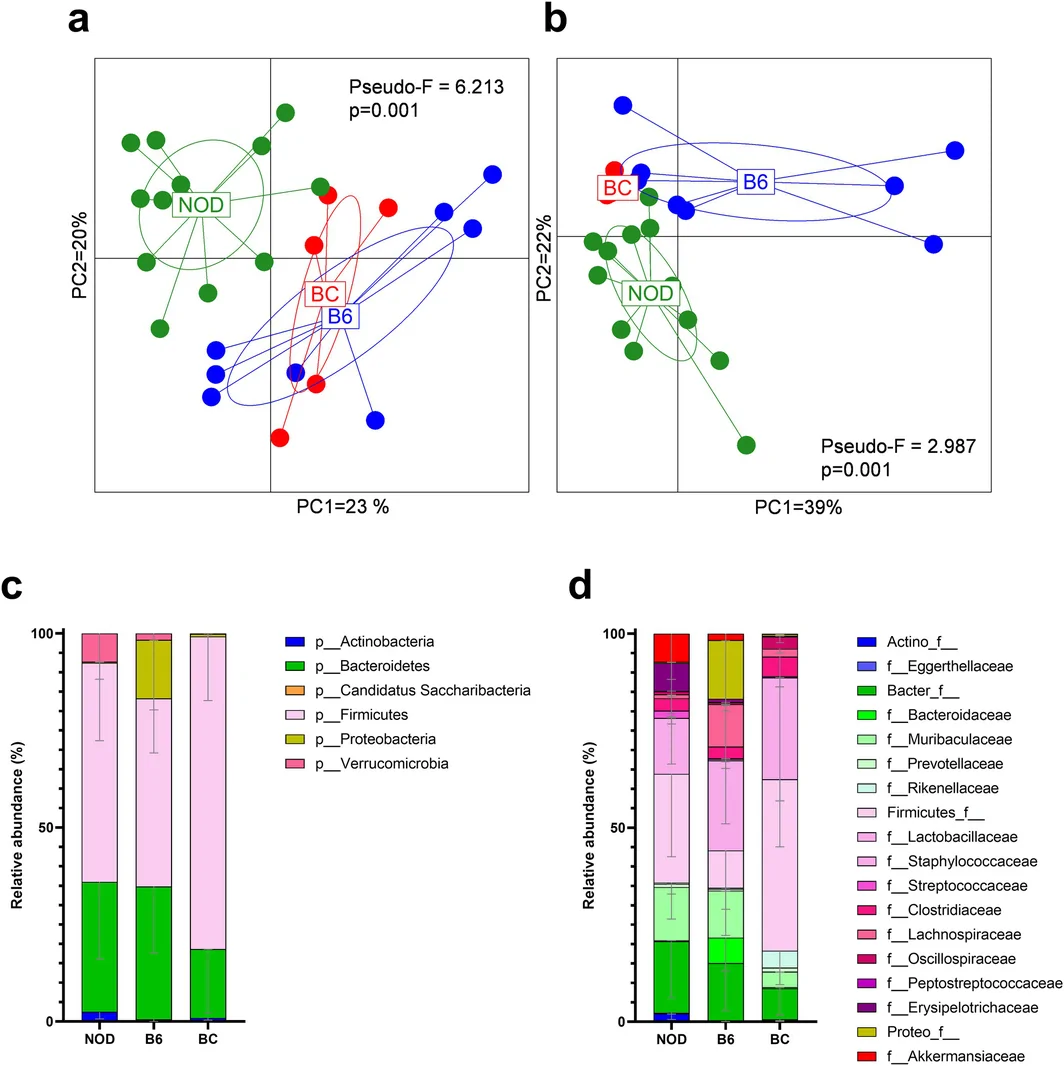
Faecal microbiota composition. Principal coordinates analysis (PCoA) plots based on Bray‐Curtis (a) and Jaccard (b) distance matrices showing qualitative and quantitative differences in the faecal microbial composition between NOD, B6 and BALB/c mice. PERMANOVA results (pseudo‐*F* and *p*) are given in the plots. Relative abundance plot of the microbial community at phylum level (c) in faecal content and at (d) family level for taxa with ≥ 1% in relative abundance in at least one sample. Green = NOD, blue = B6 (B6), red = BALB/c. *n* = NOD: 12, B6: 8, BALB/c: 5.

**FIGURE 7:**
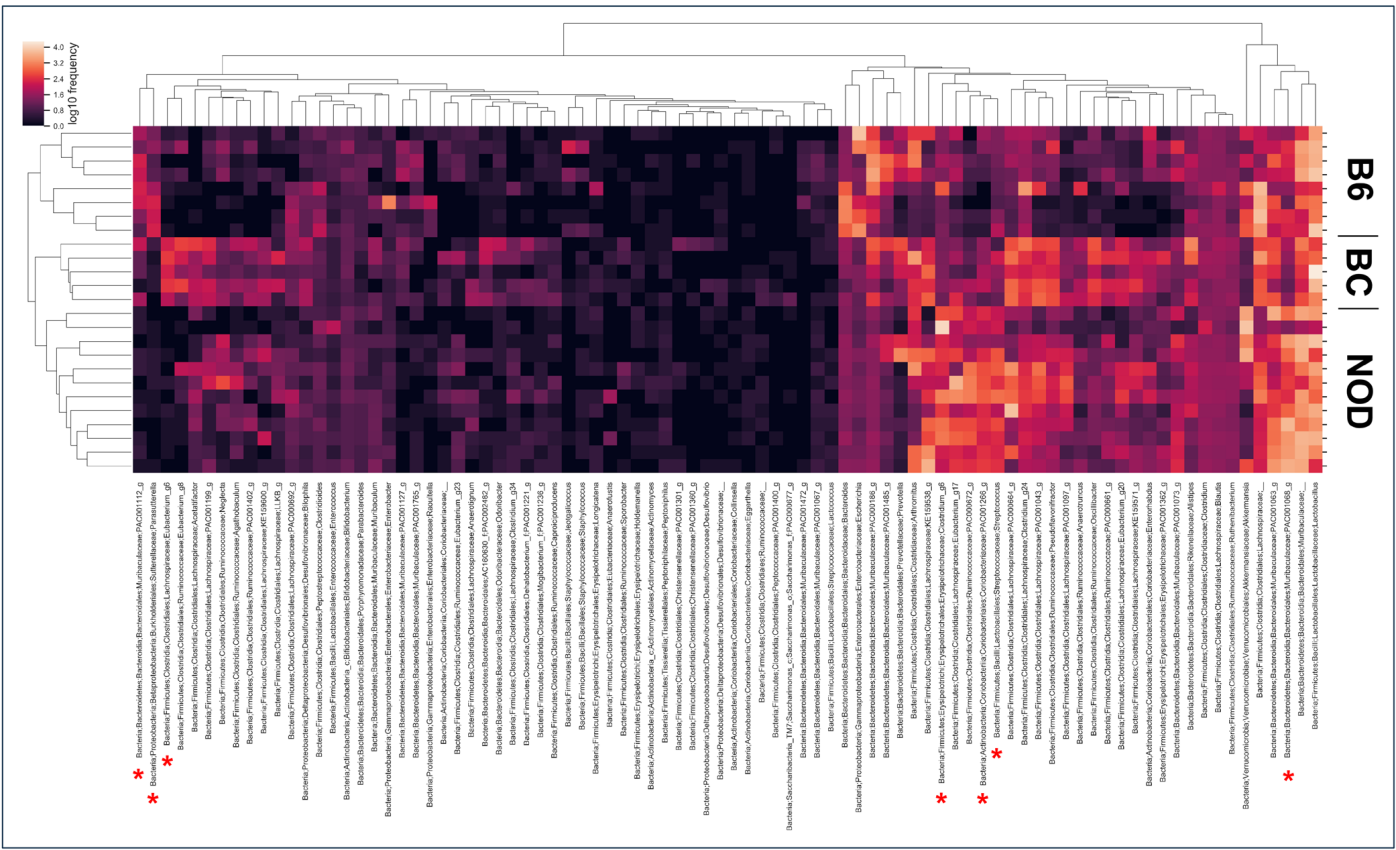
Heatmap depicting bacterial taxa relative distribution. Relative distribution of bacterial taxa depicted on log10 scale from black (log10 (0.0)) to light yellow (> log10 (4.0)) for each mouse. Bacterial taxa marked with a red * were significantly differing between the mouse strains (ANCOM, pseudo *p* < 0.05). *n* = NOD: 12, B6: 8, BALB/c: 5.

## 4 Discussion

Recently, we demonstrated reduced levels of PCs in the intestinal mucus layer of pre‐diabetic NOD mice, which may contribute to the reported intestinal barrier dysfunction in T1D. In the current study, we showed that treatment with antibiotics reduced the PC class levels in specifically the colonic mucus layer, indicating that microbiota perturbation plays a role, as hypothesized. Furthermore, our study showed that the intestinal mucus lipid composition has clear segment‐specificity. The faecal microbiota composition differed markedly between the NOD, B6 and BALB/c mice with changes in phyla including bacterial taxa known to synthesise PC, potentially explaining the observed differences in mucus PC content, although host genetics may also contribute.

Our recent study found decreased levels of the jejunal PC class in pre‐diabetic NOD compared to age‐ and diet‐matched B6 mice [10]. Furthermore, the study showed that several PC species were reduced in colonic mucus in the NOD mice. Since the cecal microbiome showed alterations between the mice in the former study, we here investigated the effect of the microbiota on the mucus PC composition. We observed segment‐specific responses of the mucus lipidome to microbiota perturbation. In the colon, antibiotic treatment reduced the abundance of the PC class in B6 and NOD mice, whereas no corresponding reduction was observed in jejunal mucus. In contrast, microbiota depletion in germ‐free BALB/c mice primarily affected the jejunum by reducing the number of detectable lipid species, while overall PC abundance remained unchanged. Thus, our data suggest that microbiota perturbation influences different lipid features in different intestinal segments rather than in a single uniform manner. The PC class reduction was not caused by reductions in specific PC species; thus, more analyses are required to unravel the mechanism behind the observed changes in PC class composition. Mucus was collected by gentle scraping, which may include epithelial cell contamination. Because all groups within each intestinal segment were processed identically, this limitation is unlikely to explain the observed strain‐ or treatment‐dependent differences, but it may influence absolute PC measurements. We were not able to replicate the recently observed reduction in jejunal PC class between pre‐diabetic NOD and B6 mice and neither on colonic PC species level, although many tended to be decreased. This may, in light of the result of the new antibiotic study, be caused by using different vendors or animal facilities in our two studies, which significantly impacts the microbial composition in the gut [30].

The reduced level of colonic mucus PC class upon antibiotics treatment may be associated with altered intestinal barrier function, which could affect, for example, bioavailability of lipid‐encapsulated colon‐released drugs [31] and lead to malabsorption of nutrients. However, this study did not directly assess intestinal barrier function or nutrient absorption. It would be relevant to test if PC‐supplementation following antibiotic treatment would alleviate gastrointestinal symptoms due to their anti‐inflammatory and mucus‐protective abilities [11, 32].

Despite the reported impact of microbiota on mucus properties [16, 33], the overall lipid compositional changes in the mucus layer were, in the current study, largely segment specific and not caused by microbiota. The observation is based on the total composition of the lipid classes and species but also on the number of detected lipid species and the PC species. The lipid profiles in jejunal and colonic mucus have previously been found spatially different in mice, dogs and pigs [34, 35, 36]. As the faecal microbial communities were also differing between NOD, B6 and BALB/c mice, this suggests that the overall mucus lipid composition is, at least not to a high degree, determined by the microbiota. These findings rather indicate that host genetic background is an important determinant of mucus lipid composition, with several strain‐dependent differences persisting independently of microbiota status. Interestingly, fully colonized BALB/c mice had the lowest colon PC level and showed the largest difference in Bacteriodetes:Firmicutes ratio including reduced abundance of Muribaculaceae; *PAC001068_g* from phylum Bacteroidetes. Bacteroidetes include PC‐containing bacteria [37], which could potentially explain the reduced PC lipid quantity in colonic mucus of these mice compared to B6 and NOD mice, and why a reduction of the bacterial load in B6 and NOD mice reduced their mucus PC level, whereas germ‐free conditions had no effect on the PC level in BALB/c mice. Hence, the effect on the mucus layer may largely have been based on which bacterial species were present and were targeted by the antibiotic treatment. Further studies are however needed to clarify whether such PC‐producing taxa contribute to host mucus PC or to what degree these differences are determined by host genetics.

In conclusion, our data demonstrate that microbiota perturbation has segment‐ and strain‐specific effects on the intestinal mucus lipidome, reducing the PC class level in the colonic mucus layer while altering the number of detected lipid species in jejunal mucus. Although further analyses are necessary, existing knowledge about PCs indicates that reduced PC levels may affect intestinal barrier function and thereby potentially influence nutrient absorption. The data also suggest that the reduced intestinal mucus PC levels that we recently reported in pre‐diabetes NOD mice may be associated with microbiota perturbation, although the present study does not distinguish between effects of altered microbial composition, reduced bacterial load or secondary host responses. In addition, the faecal microbiota composition differed between the untreated barrier‐housed mice showing changes in phyla including PC‐containing bacteria, thus potentially explaining the observed mucus PC content differences, although host genetic background may also contribute. The data showing distinct, segment‐specific mucus lipidomes in the jejunum and colon may provide valuable insights for optimizing oral drug delivery strategies.

## Author Contributions

**Mia Øgaard Mønsted:** conceptualization, methodology, formal analysis, visualization, writing – original draft. **Mesut Bilgin:** methodology, formal analysis, writing – review and editing. **Lukasz Krych:** methodology, writing – review and editing, formal analysis. **Emma Schack Wiedenbein:** methodology, writing – review and editing, formal analysis. **Camilla Hartmann Friis Hansen:** conceptualization, methodology, formal analysis, writing – review and editing. **Martin Haupt‐Jorgensen:** conceptualization, methodology, formal analysis, supervision, writing – review and editing.

## Funding

This work was funded by Poul og Erna Sehested Hansens Fond and Skibsreder Per Henriksen, R. og Hustrus Fond. The funding bodies were not involved in study design, data collection, analysis, interpretation of data, writing or submission of the article.

## Conflicts of Interest

The authors declare no conflicts of interest.

## Supporting information


**Table S1:** Lipid class distribution in jejunal and colonic mucus. Table showing the lipid composition of all detected lipid species in colonic and jejunal mucus from the mouse groups. Values are in mol%, thus the column sum equals 100.
**Table S2:** All detected lipid species in jejunal and colonic mucus. Table showing the lipid composition of all detected lipid species in colonic and jejunal mucus from the mouse groups. Values are in mol%, thus the column sum equals 100.
**Table S3:** OTU table. Table showing the abundance of operational taxonimic units (OTU) in cecal faeces from the microbiota competent mouse groups (limit ≥ 8000 reads).

## Data Availability

Lipid and microbiome data are available in the Supporting Information.
